# TrackArt: the user friendly interface for single molecule tracking data analysis and simulation applied to complex diffusion in mica supported lipid bilayers

**DOI:** 10.1186/1756-0500-7-274

**Published:** 2014-05-01

**Authors:** Artur Matysik, Rachel S Kraut

**Affiliations:** 1 School of Biological Sciences, Nanyang Technological University, 60 Nanyang Drive, Singapore 637551, Singapore

**Keywords:** Fluorescence, Single molecule tracking, Diffusion, Lipid bilayers, Total internal reflection, Microscopy, Mica, MSD

## Abstract

**Background:**

Single molecule tracking (SMT) analysis of fluorescently tagged lipid and protein probes is an attractive alternative to ensemble averaged methods such as fluorescence correlation spectroscopy (FCS) or fluorescence recovery after photobleaching (FRAP) for measuring diffusion in artificial and plasma membranes. The meaningful estimation of diffusion coefficients and their errors is however not straightforward, and is heavily dependent on sample type, acquisition method, and equipment used. Many approaches require advanced computing and programming skills for their implementation.

**Findings:**

Here we present TrackArt software, an accessible graphic interface for simulation and complex analysis of multiple particle paths. Imported trajectories can be filtered to eliminate spurious or corrupted tracks, and are then analyzed using several previously described methodologies, to yield single or multiple diffusion coefficients, their population fractions, and estimated errors. We use TrackArt to analyze the single-molecule diffusion behavior of a sphingolipid analog SM-Atto647N, in mica supported DOPC (1,2-dioleoyl-*sn*-glycero-3-phosphocholine) bilayers. Fitting with a two-component diffusion model confirms the existence of two separate populations of diffusing particles in these bilayers on mica. As a demonstration of the TrackArt workflow, we characterize and discuss the effective activation energies required to increase the diffusion rates of these populations, obtained from Arrhenius plots of temperature-dependent diffusion. Finally, TrackArt provides a simulation module, allowing the user to generate models with multiple particle trajectories, diffusing with different characteristics. Maps of domains, acting as impermeable or permeable obstacles for particles diffusing with given rate constants and diffusion coefficients, can be simulated or imported from an image. Importantly, this allows one to use simulated data with a known diffusion behavior as a comparison for results acquired using particular algorithms on actual, “natural” samples whose diffusion behavior is to be extracted. It can also serve as a tool for demonstrating diffusion principles.

**Conclusions:**

TrackArt is an open source, platform-independent, Matlab-based graphical user interface, and is easy to use even for those unfamiliar with the Matlab programming environment. TrackArt can be used for accurate simulation and analysis of complex diffusion data, such as diffusion in lipid bilayers, providing publication-quality formatted results.

## Findings

### Background

Studies on membrane dynamics using lipid- or protein-based probes specific for different membrane regions or local environments, such as domains of ordered lipids enriched in cholesterol and sphingolipids, provide deep insight into the organization of both plasma membranes and artificial lipid bilayers [1-8].

Ensemble averaging methods such as FCS or FRAP are effective for accurately determining diffusion over large data sets consisting of mobile particles. Although multiple component diffusion can be quantified using these methods, they provide limited ability to visualize single particle behaviors, such as switching between fast and slow diffusion, or confined diffusion [9,10]. Single molecule tracking (SMT) is a complementary technique that can analyze individual particle trajectories in detail, and thus is able to extract such information from recordings of diffusing particles.

For over 20 years, numerous authors have described approaches for SMT data acquisition, analysis, extraction of diffusion coefficients, and uncertainty estimation [11-15]. For each experimental paradigm, several factors that may introduce bias into the data need to be considered. The sample type and preparation (i.e. live cell, artificial membrane, mica or glass support) and the microscope setup, including the detector type, lens characteristics, labeling strategy and filter quality, all affect the signal-to-noise ratio, a crucial parameter for sensitive particle recognition and linking of detected particles into trajectories with high accuracy [16]. Acquisition parameters such as frame rate, detector gain, binning, and light intensity should be optimized. For example, a too low frame rate will result in motion- blur of moving particles. On the other hand, a too high frame rate might lead to a low signal-to-noise ratio. High light intensity increases the signal-to-noise ratio, but also results in rapid bleaching, shortening particle lifetimes (and thus trajectories). Moreover, light exposure over too long a period of time may cause sample photodamage i.e. by lipid oxidation, changing the diffusion characteristics of the sample [17]. Finally, acquired datasets must be analyzed with a view to the nature of the observed diffusion type. In both artificial and plasma membranes, diffusion is often more complex than simple Brownian diffusion in a homogenous medium, and anomalous or confined diffusion with multiple diffusing fractions are observed. This might be caused by membrane inhomogeneity, the existence of domains or impermeable clusters to the label used, or even the presence of fluorescent contaminants [13,15,18]. SMT datasets are relatively large and require initial filtering before further processing. Consequently, the final results of such analyses are extremely sensitive to the choice of proper fitting models and filtering parameters. Ideally, results on unknown data from real membranes should be supported by adequate simulations in silico. Thus, a meaningful characterization of probe dynamics requires not only good datasets, but also strong programming skills for their analysis. To simplify the process of SMT data analysis, and thereby to make it more accessible to a wide range of researchers, we designed TrackArt as a graphic user interface (GUI) implementing commonly used methods for diffusion coefficient estimation from acquired SMT data, and data filtering, as well as various modes of diffusion simulation.

Here we present features of the TrackArt interface for analysis of single molecule tracking data, and apply the workflow to describe the dynamic behavior of a fluorescently tagged sphingolipid analog, SM-Atto677N, on mica supported DOPC bilayers.

## Implementation

The TrackArt software consists of five modules: *SIM* for diffusion simulations, *IMPORT* for importing and filtering trajectories stored in an external file, *MSD* for classical MSD curve fitting, and *CPD* and *FIT* modules for resolving the *D*s of multiple subpopulations within a data set.

### Diffusion simulation

**Figure 1:**
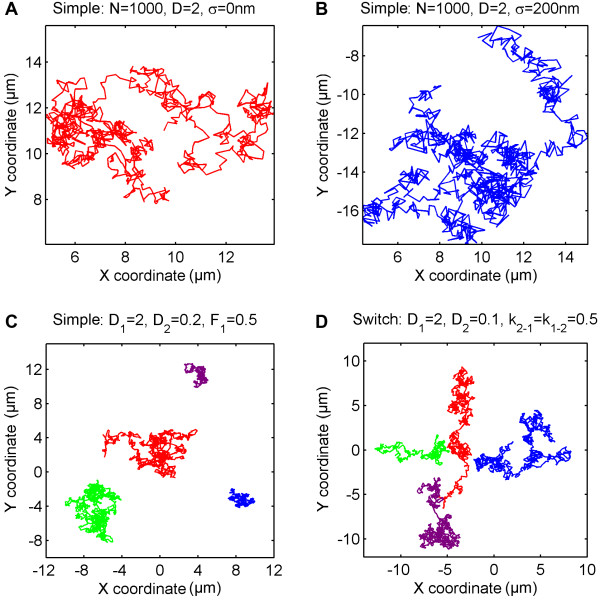
**TrackArt SIM.** Simulations in *Simple and Switch mode*. **A-B**. A single trajectory of a particle diffusing with *D* = 2 μm^2^/s, with simulated localization uncertainty σ = 0 nm and σ = 200 nm respectively. **C**. *Simple mode* simulation of four particles diffusing with *D*_*1*_ = 2 and *D*_*2*_ = 0.2 μm^2^/s, fraction = 50%. **D**. *Switch mode* simulation of four particles diffusing with *D*_*1*_ = 2 and *D*_*2*_ = 0.2 μm^2^/s. Constant rates for switch between states are equal to 0.5. For all simulations **(A-D)***N* = 1000 steps per trajectory, *Δt* = 10 ms.

As a method of validating implemented tracking data algorithms, by challenging acquired data with simulation, as well as for teaching and illustrative purposes, a simulation module was developed and included in the TrackArt ensemble. Trajectories are simulated using the Monte Carlo algorithm, for any given number of trajectories and steps, with an optional simulation of localization error (Figure 1A,B). Three simulation modes are available:

**Simple diffusion mode -** each particle moves with constant *D* throughout its lifetime. Particles can be divided into two separate populations, each moving with a different *D* (Figure 1C).

**Switching mode -** particles switch between two states, each one with a characteristic diffusion coefficients *D*_*1*_ and *D*_*2*_ with switch rates *k*_*1-2*_ and *k*_*2-1*_ (Figure 1D) according to an equilibrium:

(1) $$D_{1}\overset{k_{1-2}}{\underset{k_{2-1}}{⇄}}D_{2}$$

**Figure 2:**
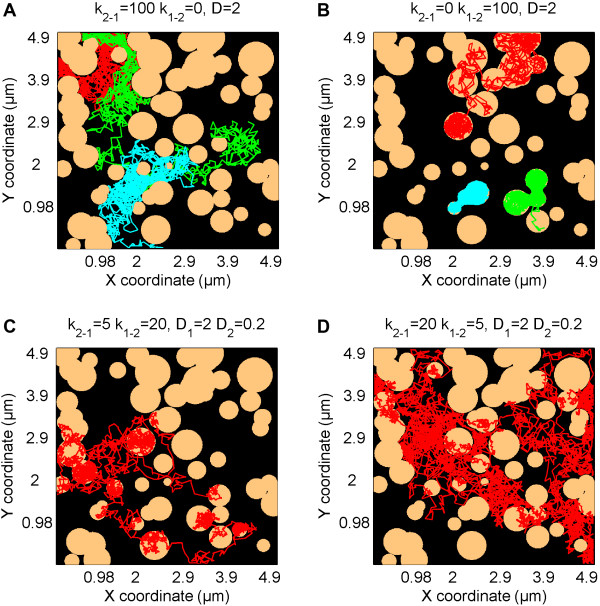
**TrackArt SIM.** Simulations in *Domain mode*. Simulations show particles behaving with different constant rates, on the same domain map. **A-B**. Trajectories of particles diffusing with *D* = 2 μm^2^/s, with *k*_*1-2*_ = 0 (domains acting as obstacles) and *k*_*2-1*_ = 0 (domains acting as region of confined diffusion), respectively. **C-D**. Particles diffusing with *D*_*1*_ = 2 and *D*_*2*_ = 0.2 μm^2^/s and constant rates **(C)***k*_*2-1*_ = 5, *k*_*1-2*_ = 20 and **(D)***k*_*2-1*_ = 20, *k*_*1-2*_ = 5. For all simulations **(A-D)***N* = 1000 steps per trajectory, *Δt* = 10 ms.

**Figure 3:**
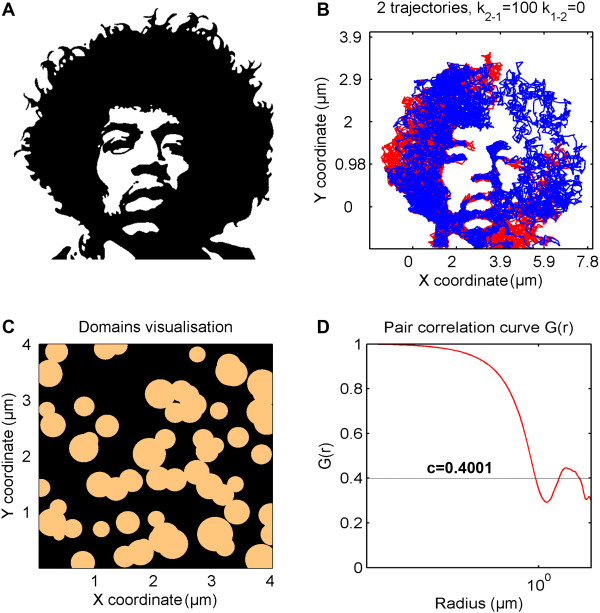
**TrackArt SIM. A**. Binary image used for import as a domain map. **B**. Simulation of two particle trajectories moving with *D* = 2 μm^2^/s, *k*_*12*_ = 0 and *k*_*21*_ = 100. *N* = 7000, *Δt* = 10 ms. **C-D**. Image of domains simulated with the domain concentration c = 0.4 (see Additional file 1) and its pair correlation function are calculated.

**Domains mode -** particles diffuse among immobile domains, simulated as circles with a given number per area, diameter, and diameter variation. The probability that a simulated particle will enter or leave a domain is regulated by constant rates *k*_*1-2*_ and *k*_*2-1*_. Depending on these constants, domains are more or less permeable to diffusing particles (Figure 2). In extreme cases, when *k*_*1-2*_ or *k*_*2-1*_ is equal to zero, domains act as impermeable obstacles, giving rise to anomalous diffusion (subdiffusion), or as permeable but viscous regions, giving rise to confined diffusion. Moreover, different diffusion coefficients can be defined for particles moving within or outside of domains. A very useful feature is that the domain map can also be imported from a binary image file, as shown in Figure 3A and 3B. This gives a user the option of running diffusion simulation on images derived from, e.g., atomic force microscopy (AFM) or other maps of actual membrane ‘landscapes’ where domains with different shapes and characteristics such as fractal dimensions, percolation, confined or hop diffusion, or grid-patterns occur. For each domain map (either simulated or imported), the pair correlation curve G(r) can also be calculated and plotted (Figure 3C,D). G(r) is the probability of finding a domain at distance *r* and allows more detailed domain map characterization [19,20].

### Data import

The import module is used to import trajectories into TrackArt from a file, such as an ImageJ plugin: Particle Tracker [21] results, or coordinates exported previously from TrackArt Simulation module. Data from other tracking software also can be imported after formatting (see Additional file 1). Once imported, one can inspect quality of tracks, and filter out unwanted trajectories, including trajectories which are too short, not linked properly, or derived from contaminants or aggregates. Filtering of imported experimental data is essential for meaningful diffusion coefficient estimation thereafter. There are several possible factors contributing to further error: samples (or surface) contamination by other fluorescent contaminants, probes forming aggregates, the existence of an immobile probe fraction (as can happen when probe sticks to a glass surface), spurious particle recognition (noise) or errors in linking coordinates into trajectories due to high particle concentration and/or fluorophore blinking. Although many of these factors can be minimized by careful surface cleaning procedures during sample preparation, the use of sensitive EMCCD cameras, high grade filters, ultra-pure lipids and solvents, and pre-bleaching of contaminants with a strong laser pulse, several exclusion criteria should be used to dismiss trajectories which may be inaccurate. In TrackArt, the aforementioned criteria are applied through various parameters: 1) a minimum number of frames required for each trajectory, 2) a minimum and maximum diffusion coefficient value calculated for each trajectory, 3) a minimum R-squared value of the linear MSD fit for each trajectory, and 4) minimum and maximum average trajectory intensity. The optimal value for each parameter is specific for the measurement conditions, equipment used, type of sample and fluorescent label. Thus, these must be determined empirically for each imaging setup. To aid in that process, TrackArt offers a wide variety of plots and data representations, such as histograms of individual *D*, trajectory length and intensity, MSD plots, and trajectory preview.

### Mean square displacement fits

Analysis of the mean square displacement (MSD) curves is the most common approach for extracting the diffusion coefficient, when only one population of particles with a single characteristic diffusion is observed. There are four models of possible MSD fits implemented in TrackArt, all of which assume particle movement in 2-dimensional space:

**Normal (Brownian) diffusion –** particles move in an isotropic medium with pure Brownian characteristics. The diffusion coefficient is extracted from the linear MSD fit:

(2) $$<r^{2}>=a+b\Delta t,\;b=4D$$

where *α* is the intercept and *t* is time. The fit can be optionally weighted by the inverse of the MSD standard deviation. In analyzing the MSD curve (and thus estimating and minimizing the error in *D*), it is important to take into account effects such as localization uncertainty, finite camera exposure and the effect of diffusion on the MSD curve. TrackArt simplifies this process by providing on output consisting of MSD standard deviation, parameter errors for weighted and unweighted fits, static and dynamic localization error, reduced localization error, error in *D* and finally the optimum number of time-lags to use for fitting (minimizing the *a* and *b* error). For these calculations, TrackArt implements algorithms and methods described in detail by Michalet et al. [11,12].

Remaining MSD fitting models include selected models described by Saxton et al. [22]:

**Anomalous diffusion (subdiffusion)** - particles diffuse among immobile obstacles, which cause deviation from Brownian diffusion;

**Free diffusion with flow** - particles undergo directional motion due to drift or active transport;

**Confined/corralled diffusion** –MSD approaches a maximum value for large time-lags, due to limitation of diffusion to within a region of confinement.

### Extraction of multiple diffusion coefficients

Analysis of MSD curves is extremely valuable for diffusion coefficient extraction. However the tracked molecules often exist as populations with different diffusion behaviors, e.g. a slow and a fast population, or a single population of particles that switch intermittently between different states. Both situations can be effectively simulated in the TrackArt simulation module. When dealing with relatively large numbers of trajectories, in both cases the overall MSD fit will provide only the mean value of *D*. Different methods of analyzing tracking data can be used to distinguish between these fractions and extract their individual *D*s [15,18,23]. The one implemented in TrackArt was proposed by Schütz et al. [15] and was successfully used in several diffusion studies [13,24-27]. Briefly, MSDs for each population and their fraction are extracted as parameters from multi-exponential fits to cumulative probability distribution (CPD) of square displacements. In TrackArt, the CPD module is used for CPD calculation and fits, whereas the FIT module calculates final values of *D*, the size of fractions diffusing with given Ds, and their errors. More detailed descriptions of each module and their algorithms can be found in Additional file 1 and user manual.

## Results and discussion

### Diffusion in a mica-supported DOPC bilayer

To show the capabilities and standard workflow of TrackArt we used single molecule data derived from diffusion of a head-labeled sphingolipid analog Sphingomyelin-Atto647N in a mica supported DOPC bilayer. We chose this probe because of its sphingolipid backbone, which might be expected to have a more complex behavior than commonly used DHPE probes.

**Figure 4:**
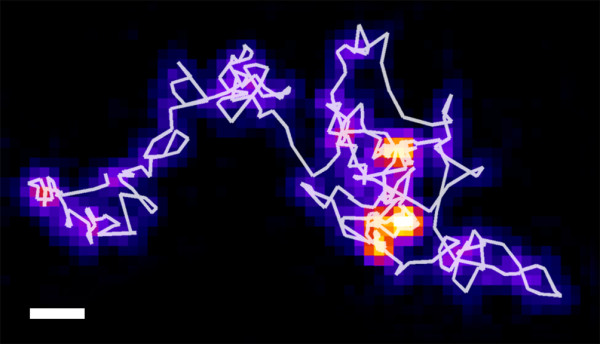
**Sum projection of a single molecule image stack, with color coding for better visualization: brighter color indicates longer time spent by the particle in a certain area.** The detected particle trajectory is overlaid on the image. It can be clearly seen that a particle is switching between two states: fast diffusion and slow diffusion. This behavior is typical for most of the observed molecules. Analyzing Particle Movement (APM_GUI) software was used to ensure that the particles remain in a confined region longer than particles undergoing normal Brownian diffusion would remain. Scale bar 1 μm.

Image stacks were acquired using TIRF microscope setup, with 10 ms time resolution at 25°C. By visual inspection, the existence of separate populations diffusing with different speeds is clearly evident. Moreover, switching between these states was often observable. An example of such a trajectory is shown in Figure 4. The summed projection (the sum of values for each pixel through the whole stack) with additional color coding was overlaid into a trajectory, to better visualize regions where the particle was residing for longer times (diffusing more slowly). To ensure that the particles remain in a confined region longer than particles undergoing normal Brownian diffusion would remain, Analyzing Particle Movement (APM_GUI) software was used [28]. Some particles were observed to be immobile for the entirety of their lifetimes, which might be attributable to membrane defects or particle adherence to the surface.

**Figure 5:**
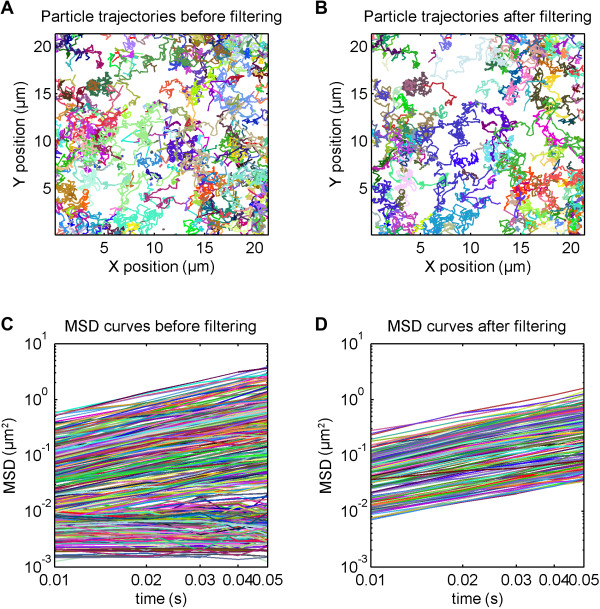
**TrackArt IMPORT. A-B**. Visualization of imported trajectories **(A)** before and **(B)** after filtering. **C-D**. MSD curves of all trajectories **(C)** before and **(D)** after filtering. Axes are in log-log scale to better visualize the filtering effect.

For detailed analysis of the diffusion behavior, first Mosaic Particle Tracker plugin [21] was used to recognize single particles and link them into trajectories. At this stage, the data must be filtered to dismiss spurious or immobile trajectories. Although this process is not complicated per se, and can be done using a variety of available programming environments or statistical software, in all cases it requires good programming skills from the user. This is especially true when dealing with large datasets, which are typical for lipid bilayer diffusion data. In TrackArt, trajectories can be easily imported, inspected and filtered. For the case of the experiment discussed above, trajectories were filtered according to criteria summarized in Table 1, and the statistics, such as number of filtered coordinates, trajectories, average *D* value and trajectory size for each dataset before and after filtering are listed in Table 2. For better visualization of the filtering process, combined trajectories and their MSD curves in log-log scale are presented in Figure 5.

**Table 1:** Exclusion criteria used for filtering spurious trajectories of SM-Atto647N diffusing on a mica-supported DOPC bilayer at 25°C

**Exclusion criterion**	**Value**
Min frames number	20
Min D (μm^2^/s)	0.1
Max D (μm^2^/s)	10
Min individual MSD fit R^2^	0.9
Min average trajectory intensity	0.3
Max average trajectory intensity	1.5

**Table 2:** Statistics of non-filtered and filtered tracking data for SM-Atto647N diffusing on a mica-supported DOPC bilayer at 25°C

	**DOPC SM-Atto647N on mica, 25°C**	
	**Non-filtered**	**Filtered**
**Coordinates**	166159	90954
**Trajectories**	2562	986
**Average trajectory length**	65	92

Datasets were then analyzed in TrackArt MSD module, by linear MSD fitting. Typically, calculation of MSDs and their linear fit to extract the diffusion coefficient can be done in a simple spreadsheet. However, a more detailed analysis returning MSD standard deviation, error in *D* (calculated using recently published algorithms [11,12]), localization error and number of optimal time-lags to use, requires more complex programming tools. Although there are direct solutions that deal effectively with the calculation of parameters mentioned [11,12], the raw code used for their implementation is daunting for those not familiar with Matlab or C++ environments. In contrast to these options, the TrackArt GUI in this example allowed easy calculation of the diffusion coefficient to 1.503 ± 0.009 μm^2^/s and the dynamic localization error to 71 nm.

**Figure 6:**
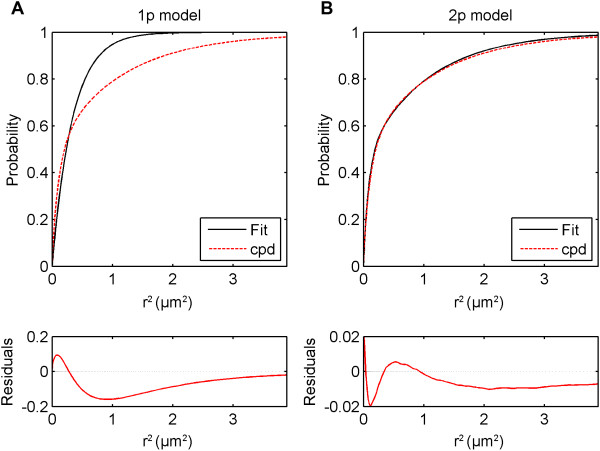
**TrackArt CPD.** Cumulative probability distribution square displacements and their fits using different models, for the 10^th^ time-lag of a DOPC-SM Atto647N sample at 25°C. **A**. A 1p model failed to fit to the data points. **B**. A 2p model successfully fit to the data points.

**Figure 7:**
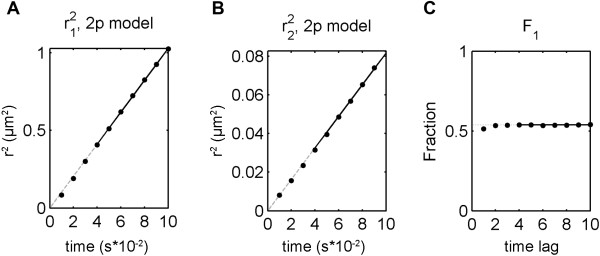
**MSD plots.** MSD plots for fast **(A)** and slow **(B)** populations, and fraction of the fast population **(C)** calculated for SM-Atto647N diffusing on a mica-supported DOPC membrane at 25°C.

Because of the aforementioned existence of multiple populations, the diffusion coefficient estimated from simple MSD fits gives only an average value of *D*. Thus, CPD and FIT modules are used to find individual *D* s of each population and their fractions. The cumulative probability distribution (CPD) of square displacements (SD) was calculated and fit in the TrackArt CPD module for the first 10 time-lags. Plots of CPD and fits aid in choosing the right model for analysis, based on residuals and calculated MSD values. As expected, a single population model failed to fit, in contrast to a two-population model (P1 and P2), which fit well (Figure 6). The three-population model did not show any fitting improvement according to an F-test and was dismissed as data over-fitting. Calculated $r_{1}^{2}$, $r_{2}^{2}$ and *F*_1_ were then plotted in the TrackArt FIT module (Figure 7). Diffusion coefficients were estimated from the linear MSD fits, while the fraction was estimated by averaging *F*_1_ parameters, resulting in: *D*_*1*_ = 2.57 ± 0.04 μm^2^/s, *D*_*2*_ = 0.20 ± 0.01 μm^2^/s and *F*_*1*_ = 53.8% ± 0.2%. The diffusion coefficient of the fast population, at 2.57 ± 0.04 μm^2^/s, is in general agreement with a recent report (2.7 ± 0.3 μm^2^/s) obtained from FCS studies on a similar mica supported bilayer [29]. In that case however, a one population model was validated and used.

A FRAP study on similar DMPC bilayers clearly indicated the existence of two populations with different diffusion rates, explained as particles diffusing in the upper and lower bilayer leaflet [30]. This explanation, however, is not consistent with our observation of switching between fast and slow states occurring in time range of seconds. Transmembrane lipid flip-flop transitions are known to occur at a rate several orders of magnitude lower [31], and will therefore be essentially unobservable. Thus, we conclude that the slowly diffusing component is more likely a result of direct interaction of individual lipid molecules with the surrounding bilayer.

### Temperature effect on diffusion

To further analyze factors controlling diffusion behavior in detail, the experiment described in the previous paragraph was repeated at four temperatures: 25°C, 30°C, 35°C and 40°C. Trajectories were filtered using the same exclusion criteria (as in Table 1). For all four conditions, CPD fits suggested the existence of two populations, and thus a 2p model was used for calculation of diffusion coefficients, which were summarized in Table 3. As expected from the Einstein relation, an increase in *D* is observed for both the fast and slow populations. Increasing error values for higher temperatures can be related to several factors. The diffusion rate increasing with temperature typically results in bigger localization errors. For the same reason, more errors are introduced during particle recognition and linking, for example because spots were below the intensity cutoff, or travelled too far between frames to be linked into the same trajectory, resulting in shorter and/or less accurate trajectories. The faster photo-bleaching rate at higher temperature may also contribute to shortening of average trajectory length. Consequently, the number of calculated squared displacements for longer time-lags was reduced drastically, increasing the final error.

**Table 3:** Diffusion coefficients and fractions for SM Atto647N diffusing on a mica-supported DOPC bilayer at different temperatures

	**Population 1**		**Population 2**
	***D***_***1 ***_**(μm**^**2**^**/s)**	***F1 *** **(%)**	***D***_***2 ***_**(μm**^**2**^**/s)**
**25°C**	2.57 **±** 0.04	53.8 **±** 0.2	0.20 **±** 0.01
**30°C**	3.24 **±** 0.04	54.2 **±** 0.4	0.28 **±** 0.01
**35°C**	3.92 **±** 0.10	53.6 ± 0.5	0.40 **±** 0.01
**40°C**	4.38 **±** 0.19	49.5 **±** 0.3	0.44 **±** 0.04

**Figure 8:**
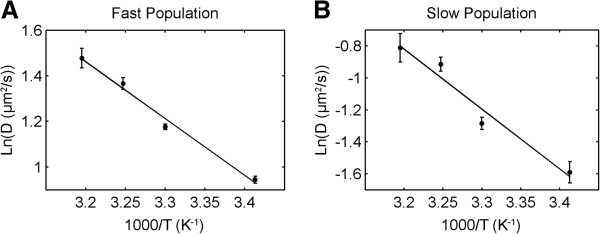
**Arrhenius plots for fast and slow populations of SM-Atto647N diffusing on a mica-supported DOPC membrane.** Activation energies calculated from the slopes for the fast **(A)** and slow **(B)** population are 30.61 ± 2.78 kJ/mol and 48.24 ± 7.20 kJ/mol respectively.

Results of diffusion at different temperatures were displayed as Arrhenius plots of *ln(D)* as a function of *1/T*, shown in Figure 8. Both populations showed highly correlated linear dependence, in perfect agreement with values expected from the Arrhenius plots, since experiments were conducted at temperatures above the DOPC melting point (*T*_*m*_ = - 20°C). The effective activation energy *E*_*Arr*_, was calculated from the slope of the least squares linear fit, yielding 30.61 ± 2.78 kJ/mol and 48.24 ± 7.20 kJ/mol for the fast and slow population, respectively. The > 1.5 times higher *E*_*Arr*_ value for the slow population supports the idea that the slower diffusion rate is due to lipid interaction with the mica surface. This would result in higher activation energy being required to overcome the surface adhesive energy, in addition to the forces from the surrounding lipids, in order to increase diffusion rate. Activation energy for the fast fraction was higher comparing to previous reports (17-20 kJ.mol) [32,33]. In those cases however, only one diffusion component was observable in bilayers supported on glass, also different lipid marker was used, and for that reason the results cannot be directly compared.

## Future directions

TrackArt can be further developed for the analysis of single molecule behavior in supported lipid bilayers and plasma membrane derived bilayers, where the role of different species of sphingolipids (e.g. complex glycolipids) on phase separation and formation of ordered domains, and the behavior of novel lipid analogs can be studied. Further, it will be interesting to characterize glycolipid- and domain- specific fluorescently tagged peptides [34] in comparison to other phase-specific probes. Indeed, the analysis of any given lipid or peptide probe in relation to a second probe or domain marker will be very informative, and can be easily achieved via the use of an emission splitting device, which records two labels simultaneously on adjacent halves of the camera chip [35]. Given its precision and sensitivity to heterogeneity in diffusion behaviors among populations of particles, TrackArt should be an effective tool for analyzing the effects of drugs and small compounds on membrane fluidity, organization, and dynamics. These studies will be done in parallel with analysis by TIRF- and confocal-FCS methods [36-39], which are complementary to SMT.

## Conclusions

SMT is a commonly used and effective technique for analysis of diffusion in lipid bilayers, and while it is traditionally viewed as being overly labor-intensive for the extraction of very precise diffusion coefficients from large data sets, the method provides different and complementary information to other statistical methods such as FCS or FRAP. Most importantly, SMT allows direct observation and measurement of individual molecules in a lipid membrane, and because of this it can give insights into the behavior of individuals with respect to their environment. However, the accurate quantitative analysis of diffusion data requires a careful approach that includes trajectory filtering, as well as selection of proper models, and methods of analysis. Although many methods have been described and effectively used for ~20 years, their application often assumes good programming skills, and their adoption by the non-specialist community is hampered by the lack of easy to use tools. In this manuscript, we presented the TrackArt software ensemble for SMT data analysis and simulation. As an example of its effectiveness, we used TrackArt to analyze and describe the complex diffusion behavior of a sphingolipid analog in mica-supported DOPC bilayers. Encouragingly, we were able to detect in this system the existence of different diffusion components, their dependence on temperature, and finally their effective activation energies, suggesting transient interactions of the slower-diffusing molecules with the mica surface. The graphical interface of TrackArt allows fast and accurate SMT data processing, even for those not familiar with any programming environment, and the workflow can be repeated on test data provided. TrackArt will help to achieve the goal of making a very useful, but sometimes esoteric and tricky technique more accessible to the biological community.

## Availability and requirements

• **Project name:** TrackArt

• Project home page:

• http://www2.sbs.ntu.edu.sg/staff/rskraut/index.php/trackart

• http://sourceforge.net/projects/trackart/

• **Operating system(s):** Platform independent

• **Programming language:** Matlab

• **Other requirements:** none

• **License:** GNU General Public License

• **Any restrictions to use by non-academics:** none

## Abbreviations

AFM: Atomic force microscopy; CPD: Cumulative probability distribution; DOPC: 1,2-dioleoyl-*sn*-glycero-3-phosphocholine; EMCCD: Electron multiplying charge coupled device; FCS: Fluorescence correlation spectroscopy; FRAP: Fluorescence recovery after photobleaching; GUI: Graphic user interface; MSD: Mean square displacement; SMT: Single molecule tracking; TIR: Total internal reflection.

## Competing interest

The authors declare that they have no competing interests.

## Authors’ contributions

AM and RK conceived and designed this study. AM carried out experiments and designed and coded the software. AM and RK wrote the manuscript. Both authors read and approved the final manuscript.

## Supplementary Material

Additional file 1 TrackArt: the user friendly interface for single molecule tracking data analysis and simulation applied to complex diffusion in mica supported lipid bilayers - supplementary materials. Click here for file
