# Supplementary material for: TrackArt: the user friendly interface for single molecule tracking data analysis and simulation applied to complex diffusion in mica supported lipid bilayers
Source: BMC Res Notes. 2014 May 1;7:274. doi: 10.1186/1756-0500-7-274 (PMC4021860; doi:10.1186/1756-0500-7-274)
Supplement: Additional file 1 — TrackArt: the user friendly interface for single molecule tracking data analysis and simulation applied to complex diffusion in mica supported lipid bilayers - supplementary materials. [file 1756-0500-7-274-S1.doc]

Supporting Material

### TrackArt: the user friendly interface for single molecule tracking data analysis and simulation applied to complex diffusion in mica supported lipid bilayers

Artur Matysik^*^, Rachel S. Kraut^**^

School of Biological Sciences, Nanyang Technological University,
60 Nanyang Drive, 637551, Singapore

E-mail:^*^ artur1@e.ntu.edu.sg, ^**^ rskraut@ntu.edu.sg

# TrackArt installation and requirements

## Requirements:

TrackArt is the graphic user interface written in a Matlab programming environment. It is platform independent and was tested on Matlab 7.10.0 (R2010a).

## Running TrackArt:

The current version of TrackArt can be downloaded from:
<http://www2.sbs.ntu.edu.sg/staff/rskraut/index.php/trackart/download>. To run TrackArt, unzip the package, add the TrackArt folder to the Matlab path, including its subfolders and type trackart in the Matlab command line.

# Data import.

TrackArt was designed to import result files from the Mosaic Particle Tracker Imagej plugin . The result file is obtained by selecting *“All trajectories to Table”* in the plugin *“Results”* window and saving data opened in new window (*File > Save as…*) and has the 12 column *TAB* delimited format:

- (column 1) – coordinate number
- (column 2) – trajectory number
- (column 3) – frame number
- (columns 4-5) – *X* and *Y* coordinates
- (column 6) – *Z* coordinate (for 3D tracking, not used by TrackArt)
- (columns 7-11) – intensity moments at the detected locations of order 0 to 4 (used by TrackArt to calculate the mean trajectory intensity
- (column 12) – NPscore (not used by TrackArt)

Source data from different tracking software can be also used with TrackArt. They need to be however reformatted to coma separated values file type (*.csv) containing the following comma separated values in the each row:

1. **Absolute trajectory number**
2. **Dataset number** (use value 1)
3. **Trajectory number within the dataset** (use the same value as in #1)
4. **Frame number**
5. **X –coordinate** (given in pixels or in μm)
6. **Y-coordinate** (given in pixels or in μm)

# Simulations

## Simple mode

Simulated particles undergo Brownian diffusion with diffusion coefficient $D_{1}$or$D_{2}$, with fraction $F_{1}$and $F_{2}$ respectively. Because$F_{2}=1-F_{1}$, only $F_{1}$ is used in calculations. Particles diffuse with the same $D$for the whole lifetime. When the fraction multiplied by the number of simulated particles is not an integer (i.e. three particles and an equal fraction of$F_{1,2}=0.5$), the number of particles diffusing with$D_{1}$ is rounded to the nearest integer.

## Switch mode

Every simulated particle undergoes Brownian diffusion and switches between diffusion coefficients $D_{1}$and $D_{2}$ with switch rates k_1-2_ and k_2-1_ according to an equilibrium:

The probability of switching within time $\Delta t$is defined as:

$$\begin{matrix} p_{21}=\Delta tk_{2-1} \\ p_{12}=\Delta tk_{1-2} \end{matrix}$$

The average time in the first state relative to particle lifetime is defined as fraction ($F_{1}$):

$$F_{1}=\frac{k_{2-1}}{k_{2-1}+k_{1-2}}$$

As previously, $F_{2}=1-F_{1}$. The actual fraction, however, may vary from the theoretical value due to the stochastic nature of the simulation, and is calculated as:

$$F_{1,actual}=\frac{states D_{1}}{N}$$

where *states D_1_* is the number of steps when a particle is in the first state, and *N* is the total step number.

## Domains mode

### Diffusion simulation

In this mode, each particle diffuses over a simulated or imported domain map. Depending on the presence or absence of a domain at the current particle position (referred to hereafter as particle state), the particle moves with constant $D_{1}$or$D_{2}$, respectively.

When the particle approaches a domain (or is about to escape from one), i.e. when the next simulated coordinate will cause a change in particle state, the move will be accepted with a probability defined similarly to :

$$\begin{matrix} p_{21}=\Delta tk_{2-1} (domain exit) \\ p_{12}=\Delta tk_{1-2} (domain enter) \end{matrix}$$

In case the move is rejected, another set of coordinates is drawn and verified. If $k_{1-2}$or $k_{2-1}$are set to zero, it is impossible for a particle to move inside or outside the domain, thus causing them to act like obstacles (anomalous diffusion) or confining regions (confined diffusion), respectively.

The average time in the first state relative to particle lifetime is calculated as in switch mode .

### Localization error

Localization error is simulated by adding to every coordinate a pseudo-random value drawn from the standard normal distribution, multiplied by a desired σ:

$$\begin{matrix} x_{err}=x_{0}+randn_{x}\cdot\sigma\\ y_{err}=y_{0}+randn_{y}\cdot\sigma\end{matrix}$$

where $x_{err},y_{err}$and $x_{0},y_{0}$ are coordinates with and without localization error, $randn$is a normally distributed (with the mean equal to zero) pseudo-random value, generated separately for $x_{0}$ and $y_{0}$, and σ is desired localization error.

### Domain map simulation

Domains are simulated as circles of given radius$r_{0}$. Additional variation in domain size might be introduced using a *variation* parameter$v$, as the multiplication of normally distributed random values $randn$added to the radius$r_{0}$:

$$r=r_{0}(1+v\cdot randn)$$

Domains are simulated iteratively, until the desired concentration $c$is reached. $c$ is defined as the domain area divided by the total map area.

# MSD Module

Normal (Brownian) diffusion

### Diffusion coefficient

Particles move in an isotropic medium with pure Brownian characteristics. The diffusion coefficient $D$ is extracted from the linear MSD fit:

$$<r^{2}> =a+b\Delta t$$

as:

$$D=b/4$$

where$a$and $b$are the parameters.

### Localization uncertainty

Dynamic localization uncertainty is calculated as :

$$\sigma=\frac{1}{2}\sqrt{a+\frac{bt_{E}}{3}}$$

where $t_{E}$is the camera exposure time.

Static localization uncertainty is calculated as:

$$\sigma_{0}=\frac{\sigma s_{0}}{\sqrt{s_{0}^{2}+\frac{bt_{E}}{4}}}$$

where $s_{0}$is the point spread function (PSF) size. $s_{0}$ can be estimated experimentally (i.e. using “PSF 2D tool” ImageJ plugin ) or calculated as described by Michalet et al. 2010 :

Reduced localization error $x$is calculated as:

$$x=\frac{\varepsilon}{\alpha}$$

where:

$$\varepsilon=a+\frac{4}{3}Dt_{E}$$

and

$$\alpha=b\Delta t$$

### MSD standard deviation

MSD standard deviation is calculated as:

$$\begin{matrix} f(n,N,x)^{-1}=\frac{n}{6K^{2}}(4n^{2}K+2K-n^{3}+n)+\frac{1}{K}(2nx+(1+\frac{1}{2}(1-\frac{n}{K}))x^{2}); n\leq K \\ f(n,N,x)^{-1}=\frac{1}{6K}(6n^{2}K-4K^{2}+4n+K^{3}-K)+\frac{1}{K}(2nx+x^{2}); n>K \end{matrix}$$

In the calculations of Qian and Michalet , *K* is defined as
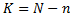
, where *N* is the number of steps and n is the time-lag. This is however valid only for single trajectory considerations. When analyzing SMT data however, trajectories of different length are pooled and analyzed together. Thus, the *K* value in TrackArt is treated as the total number of displacements calculated for time-lag *n*.

### Error in D

The relative error in *D* is calculated according to Qian et al. 1991 :

$$\rho=\sqrt{2m/3K}$$

where $m$defines the number of time-lags to fit. This equation however does not include localization error and although it is used in numerous publications, it underestimates error . More accurate error estimation requires a determination of reduced localization error, then MSD standard deviation and covariance, and finally errors of $a$and $b$fit parameters. All these procedures are also implemented in TrackArt and displayed on output. However, the full equations are not included here as they are complex, and can be found with a detailed explanation in Michalet et al. .

### Calculation of optimal time-lags to fit (P_min_):

The optimal number of time-lags to fit is calculated using a simplified algorithm described by Michalet et al. . First, the linear MSD fit is calculated to number of time-lags defined by the *Lags to show* parameter. Then, a reduced localization error is estimated:

$$x_{0}=\frac{4\sigma_{0}^{2}}{bt}$$

where:

$\sigma_{0}$is the static localization error calculated or estimated experimentally.

Then$P_{\min,1}$ is calculated as:

$$P_{\min,1}=E(2+2.7x_{0}^{0.5})$$

Where:

$x_{1}$is the first estimation of reduced localization uncertainty.

Finally, another linear MSD fit is conducted for a calculated number of lags ($P_{\min,1}$), and a new estimation of reduced localization error is calculated as:

$$x=\frac{a}{bt}$$

$P_{\min,i}$is then re-calculated using the new $x$ value. This process is repeated until no further change in$P_{\min}$occurs.

## Other diffusion models

Other diffusion models implemented into TrackArt are anomalous diffusion, diffusion with flow and confined/corralled diffusion, as described by Saxton et al. :

### Anomalous diffusion (subdiffusion)

Particles diffuse among immobile obstacles, resulting in deviation from Brownian diffusion. The MSD curve has the form:

$$<r^{2}> =a+4\Gamma t^{\alpha} \alpha<1$$

where $\alpha$ is a parameter differentiating anomalous from normal diffusion and *Γ* is a transport coefficient (note, that for $\alpha=1$, the equation describes normal diffusion, and *Γ=D*).

### Free diffusion with flow

Particles undergo directional motion due to drift or active transport. The MSD curve can be described and fit as:

$$<r^{2}> =a+v^{2}t^{2}+4Dt$$

where $v$ is the velocity of directed motion.

### Confined / corralled diffusion

The MSD approaches a maximum value for large $t$ due to limitation of diffusion within a confinement region, and has the form:

$$<r^{2}> =a+<r_{c}^{2}>[1-A_{1}\exp(-A_{2}4Dt/<r_{c}^{2}>)]$$

where $<r_{c}^{2}>$ is the confinement size and $A_{1},A_{2}$ describe confinement geometry.

# CPD and FIT Module

The cumulative probability distributions (CPD) of squared displacements,
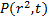
, are calculated for a given number of time-lags and then fitted to three equations, representing one- two- and three-population models, using the least squares method :

$$P(r^{2},t)=1-\exp^{\frac{-r^{2}}{r_{0}^{2}}}$$

$$P(r^{2},t)=1-\left[F_{1}\cdot\exp^{\frac{-r^{2}}{r_{1}^{2}}}+(1-F_{1})\cdot\exp^{\frac{-r^{2}}{r_{2}^{2}}}\right]$$

$$P(r^{2},t)=1-\left[F_{1}\cdot\exp^{\frac{-r^{2}}{r_{1}^{2}}}+F_{2}\cdot\exp^{\frac{-r^{2}}{r_{2}^{2}}}+F_{3}\cdot\exp^{\frac{-r^{2}}{r_{3}^{2}}}\right]; \mathrm{F}_{1}+\mathrm{F}_{2}+\mathrm{F}_{3}=1$$

Prior to fitting, CPDs can be optionally binned and filtered, by setting an additional cutoff for the $P$ or $r^{2}$ values. Additionally, any fit parameter can be fixed at a given value. Plots of CPD, their fits with residuals and $r_{i}^{2}(t)$ plots allow easy selection of the right CPD fitting model. Diffusion coefficients are calculated from slope of linear fits $r_{i}^{2}$vs. time, as in the standard MSD method, with error$D\sim\pm\sqrt{2m/3K}$. The fraction of each population is estimated by averaging over a selected time-lag region, with standard deviation as error.

# Test data

## About the test data

The test data contains unfiltered, linked trajectories of SM-Atto647N molecules diffusing in a mica supported DOPC bilayer at four different temperatures: 25, 30, 40 and 45°C. The same dataset was analyzed and described in this manuscript.

## Materials and methods

### Chemicals:

DOPC (1,2-dilauroyl-sn-glycero-3-phosphocholine) was from AVANTI Polar lipids (Alabaster, AL). Head labeled sphingolipid SM-Atto647N (N-Atto647N-sphingosylphosphocholine was from Atto-Tec (Siegen, Germany).

### SLB formation:

Appropriate volumes of DOPC and SM-Atto647N in chloroform were mixed in a round bottom flask (0.4 mg of DOPC, 0.003 mol% of SM-Atto647N). Chloroform was evaporated using a rotary evaporator (Rotavap R-210, Buchi, Switzerland) for 2 hours at 65°C. The lipid film was re-suspended in 1ml HBSS / 10mM HBSS, pH 7.4, vortexed 10 min, then freeze – thawed 5 times. The solution was then extruded through a membrane with 50nm pore size (Whatman, Clifton, NJ) using an extruder syringe (Hamilton Company, USA). 20μl of such a vesicle solution was deposited onto a freshly cleaved mica surface (SPI Supplies, USA), which was glued to a 0.17mm coverslip with optical glue (Norland Products Inc., USA) and mounted onto a custom made plastic holder. After 30 min of incubation at room temperature, the membrane was washed 10x with the same HBSS/10mM HEPES pH 7.4 buffer, to remove unfused vesicles.

### Data acquisition:

For the total internal reflection microscopy (TIRF) imaging, a modified Nikon Eclipse TE2000U TIRF setup was used, equipped with a 100x 1.49NA objective (Nikon, Japan). Laser light from a 638nm LED laser was passed through a 660LP dichroic mirror (Chroma Technology Corp, Bellows Falls, VT) and used for sample illumination under the TIR angle. Emitted light was filtered using a 738/75 excitation filter and collected on a back-illuminated EMCCD camera chip (Andor iXON897, 512x512px, Andor Technology, USA). The temperature of the sample was regulated using aTokai Hit INU heating stage and a controller for cell measurements (Tokai Hit Co., Ltd, Japan), and the actual temperature of the sample was verified continuously using a thermocouple. Data was acquired at four temperatures: 25, 30, 35 and 40°C. After each temperature adjustment, the sample was left for 30min to equilibrate. The image acquisition software was NIS-Elements (Nikon, Japan). Typically, for each measurement 4000 frames were collected with 10ms exposure time and stored in the NIS-Elements format. For each temperature condition, 15 movies were acquired, each from a different area of the sample.

### Image Processing:

Acquired stacks were first converted to 16-bit Tiff files using the ImageJ plugin BioFormats . The Mosaic Background Subtractor ImageJ plugin was used to subtract background artifacts due to uneven illumination. The Mosaic Particle Tracker ImageJ plugin was then used to localize particles and link them into trajectories. Results were exported to a table, and then saved to a file.

# References

1. Sbalzarini IF, Koumoutsakos P: **Feature point tracking and trajectory analysis for video imaging in cell biology**. *J Struct Biol* 2005, **151**(2):182-195.

2. Linkert M, Rueden CT, Allan C, Burel JM, Moore W, Patterson A, Loranger B, Moore J, Neves C, Macdonald D *et al*: **Metadata matters: access to image data in the real world**. *J Cell Biol* 2010, **189**(5):777-782.

3. Michalet X: **Mean square displacement analysis of single-particle trajectories with localization error: Brownian motion in an isotropic medium**. *Phys Rev E Stat Nonlin Soft Matter Phys* 2010, **82**(4 Pt 1):041914.

4. Michalet X, Berglund AJ: **Optimal diffusion coefficient estimation in single-particle tracking**. *Phys Rev E Stat Nonlin Soft Matter Phys* 2012, **85**(6 Pt 1):061916.

5. Qian H, Sheetz MP, Elson EL: **Single particle tracking. Analysis of diffusion and flow in two-dimensional systems**. *Biophys J* 1991, **60**(4):910-921.

6. Saxton MJ, Jacobson K: **Single-particle tracking: applications to membrane dynamics**. *Annu Rev Biophys Biomol Struct* 1997, **26**:373-399.

7. Schutz GJ, Schindler H, Schmidt T: **Single-molecule microscopy on model membranes reveals anomalous diffusion**. *Biophys J* 1997, **73**(2):1073-1080.
